# Exploring Social Media Abuse in Nursing Homes: A Convert Form of Elder Abuse

**DOI:** 10.1093/geroni/igaf122.3931

**Published:** 2025-12-31

**Authors:** Wei-Lin Xue, Eilon Caspi, Layana Aoun, Christy Aoun, Pi-Ju Liu

**Affiliations:** Case Western Reserve University, Cleveland, Ohio, United States; University of Minnesota, Minneapolis, Minnesota, United States; Purdue University, West Lafayette, Indiana, United States; Purdue University, West Lafayette, Indiana, United States; Purdue University, West Lafayette, Indiana, United States

## Abstract

Elder abuse in nursing homes manifests in various forms, including physical harm by staff and neglect of resident care. A newly emerging form of abuse and privacy violation, social media abuse, has become increasingly prevalent but remains largely unrecognized. Social media abuse involves the unauthorized taking and posting of photos and/or videos of residents on platforms such as Snapchat, Facebook, and TikTok, often accompanied by demeaning or abusive contents. Social media abuse in nursing homes has not been systematically examined, with little understanding of characteristics and impact of this type of abuse on residents. This exploratory qualitative study utilizes de-identified data (state investigation reports) from 100 nursing homes across 31 states, accessed through the Nursing Home Inspect (ProPublica) website, to understand social media abuse in nursing homes. Preliminary analysis of investigation reports reveals evidence of privacy violations and abuse affecting 152 nursing home residents. Approximately 85% of these victims exhibited some degree of cognitive impairment, with at least 50 residents suffering from moderate-to-severe cognitive deficits. The findings aim to inform care practices and policy changes to protect residents’ privacy, dignity and safety.

